# Augmented reality for stroke rehabilitation during COVID-19

**DOI:** 10.1186/s12984-022-01100-9

**Published:** 2022-12-08

**Authors:** Zhen-Qun Yang, Dan Du, Xiao-Yong Wei, Raymond Kai-Yu Tong

**Affiliations:** 1 Department of Biomedical Engineering, The Chinese University of Hong Kong, Hong Kong SAR, China; 2 Department of Computer Science, Sichuan University, Chengdu, China

**Keywords:** Stroke rehabilitation, Augmented reality (AR), COVID-19, Human–machine integrated training

## Abstract

**Background:**

The lack of the rehabilitation professionals is a global issue and it is becoming more serious during COVID-19. An Augmented Reality Rehabilitation System (AR Rehab) was developed for virtual training delivery. The virtual training was integrated into the participants’ usual care to reduce the human trainers’ effort so that the manpower scarcity can be eased. This also resulted in the reduction of the contact rate in pandemics.

**Objective:**

To investigate the feasibility of the AR Rehab-based virtual training when integrated into the usual care in a real-world pandemic setting, by answering questions of whether the integrated trials can help fulfill the training goal and whether the trials can be delivered when resources are limited because of COVID-19.

**Methods:**

Chronic stroke participants were randomly assigned to either a centre-based group (AR-Centre) or a home-based group (AR-Home) for a trial consisting of 20 sessions delivered in a human–machine integrated intervention. The trial of the AR-Centre was human training intensive with 3/4 of each session delivered by human trainers (PTs/OTs/Assistants) and 1/4 delivered by the virtual trainer (AR Rehab). The trial of the AR-Home was virtual training intensive with 1/4 and 3/4 of each session delivered by human and virtual trainers, respectively. Functional assessments including Fugl-Meyer Assessment for Upper Extremity (FMA-UE) and Lower Extremity (FMA-LE), Functional Ambulation Category (FAC), Berg Balance Scale (BBS), Barthel Index (BI) of Activities of Daily Living (ADL), and Physical Component Summary (SF-12v2 PCS) and Mental Component Summary (SF-12v2 MCS) of the 12-Item Short Form Health Survey (SF-12v2), were conducted before and after the intervention. User experience (UX) using questionnaires were collected after the intervention. Time and human resources required to deliver the human and virtual training, respectively, and the proportion of participants with clinical significant improvement were also used as supplementary measures.

**Results:**

There were 129 patients from 10 rehabilitation centres enrolled in the integrated program with 39 of them were selected for investigation. Significant functional improvement in FMA-UE (AR-Centre: *p* = 0.0022, AR-Home: *p* = 0.0043), FMA-LE (AR-Centre: *p* = 0.0007, AR-Home: *p* = 0.0052), SF-12v2 PCS (AR-Centre: *p* = 0.027, AR-Home: *p* = 0.036) were observed in both groups. Significant improvement in balance ability (BBS: *p* = 0.0438), and mental components (SF-12v2 MCS: *p* = 0.017) were found in AR-Centre group, while activities of daily living (BI: *p* = 0.0007) was found in AR-Home group. Contact rate was reduced by 30.75–72.30% within AR-All, 0.00–60.00% within AR-Centre, and 75.00–90.00% within AR-Home.

**Conclusion:**

The human–machine integrated mode was effective and efficient to reduce the human rehabilitation professionals’ effort while fulfilling the training goals. It eased the scarcity of manpower and reduced the contact rate during the pandemics.

## Background

**Fig. 1:**
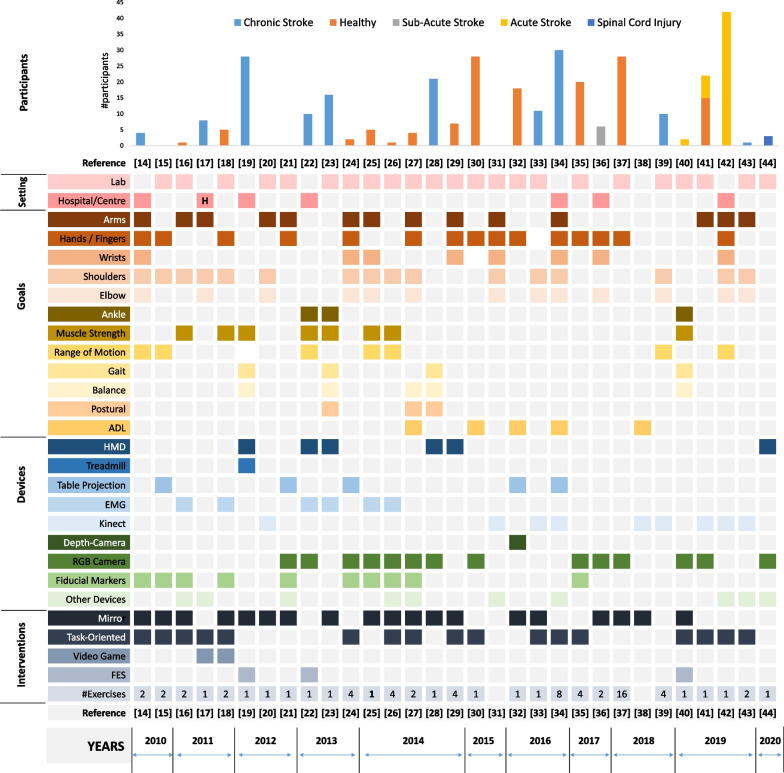
AR rehabilitation studies/systems in recent 10 years. *Lab* lLaboratory, *H* home, *ADL* activity of daily living, *HMD* head-mounted display, *EMG* electromyography, *FES* functional electric stimulation

Stroke is the second commonest cause of death and one of the leading causes of disability [1–3]. Researches show that most of these deaths can be prevented through early interventions with rehabilitation training programs [4]. However, this requires intensive participation of physiotherapists (PTs), occupational therapists (OTs), and rehabilitation assistants, which are the scarce resources in most of the countries/regions [4–6]. In Hong Kong, patients are facing the similar scarcity, indicated by the statistics of Strategic Review on Healthcare Manpower Planning and Professional Development of Hong Kong in 2017 [7] in which the manpower gap of PTs (OTs) is 17% (6.6%) in 2016 and will gradually increase to 26.8% (11.2%) by 2030 because of the large ageing population (1.40 million or 18.3% of the total population of age 75 or above is predicted). However, the situation is becoming even worse since 2020 as a consequence of the COVID-19 [8, 9]. Rehabilitation centres limited their service to reduce the contact rate so as to reduce the risk of infections of this most vulnerable group of populations. In a period from February 2020 to February in 2021, many rehabilitation centres were suspended because of the first to fourth waves of pandemic [10, 11]. Many patients have missed their golden period of the rehabilitation [12]. The traditional and manpower intensive rehabilitation mode is challenged by both the scarcity of human resources and the strict regulations for contact rate reduction [13]. The emerging technology of Virtual Reality (VR) or Augmented Reality (AR), which has been validated as a promising way to assist the rehabilitation training in previous studies [14–46], has a great potential to save the efforts of rehabilitation professionals so as to ease the scarcity issue. The study of VR/AR stroke rehabilitation can date back to 2004, when Jaffe et al. built a VR training environment with a motorised treadmill, a head-mounted display (HMD), and shoes with switches on the bottom for training the patients to step over the virtual obstacles. The experimental results provided evidence with enhanced clinical performance [45]. Later on, many systems have been developed and reported with encouraging results for stroke recovery. The most recent work include the AR-augmented wheelchairs by Daniel et al. in 2020 [44], the Kinect-based training by Adyasha et al. [41] and Au${\check{s}}$ra et al. [42] in 2019. In Fig. 1, we have summarized the representative AR stroke rehabilitation studies/systems in recent 10 years. We can see that a diverse range of studies/systems have been conducted/built. Some are for the upper extremity including those for arms [14, 16, 17, 20, 21, 24, 25, 27, 29, 31, 34, 41–43], hands/fingers [14, 15, 18, 21, 24, 27, 29–32, 34–37, 42], and shoulders [14–18, 20, 24–27, 31, 33, 34, 39, 42, 43], while the others are on the lower extremity including those for ankles [22, 23, 40], gait [19, 23, 28, 40], balance [19, 23, 27, 28, 40], and postural [23, 27, 28]. However, most of these studies are conducted in laboratory setting and thus are less feasible in real clinical scenarios^1^.

First of all, those studies focus on the specific aspects of the recovery for research purpose (e.g., 24 of them are for upper extremity, 8 for muscle strength, and 4 for gait), while in a real clinical setting, the training needs to be designed as practical programs which are comprehensive enough to cover not only as many parts as necessary, but also the whole-body coordination and the quality of life. Furthermore, those systems may require complicated ad-hoc environmental setup which are not practical in rehabilitation centres or hospitals. For example, a precise camera calibration has to be done prior to the mirror training [14–16, 18–21]. Some systems require the patients to wear the HMD during training [19, 22, 23, 28, 29] which is with limited acceptability to patients, not to mention that most of the post-stroke patients are with balance impairments and it is dangerous for them to wear HMD during training. Moreover, none of previous studies has provided a quantitative analysis on how the AR systems can help to reduce the rehabilitation manpower while fulfilling the training goals, which is critical to rehabilitation centres and hospitals. It is especially important during COVID-19 pandemics.

The purpose of this study is to investigate the feasibility of the AR rehabilitation system in a real-world setting, in which it is used as a part of an integrated training program of the usual care. The setting of the integrated mode rather than applying AR training alone (like in most of the previous studies) is due to a conclusion made by Kate et al. [47]. Based on the survey including 72 trials of 2470 participants, Kate et al. concluded that the AR/VR for stroke rehabilitation, when was used alone, was not more beneficial than conventional therapy approaches in improving upper limb functions, but it could make a statistical difference when used as an adjunct to usual care [36]. Our study follows this thread. Furthermore, the study has been conducted under the emergency situations of COVID-19 in Hong Kong when the demand for reducing the manpower and contact rate reached the pandemic level. The following questions were addressed: (a) can the integrated program fulfil the training goals when a considerable part of the training (25 to 75%) has been replaced with AR guided training? (b) how much and what types of manpower can be saved while maintaining the efficacy of training with the assistance of the AR rehabilitation? (c) how can the contact rate be reduced with the AR training? (d) how do therapists/patients experience the AR training?

## Methods

The study was an assessor-blind, randomized controlled trial and was approved by the Joint Chinese University of Hong Kong—New Territories East Cluster Clinical Research Ethics Committee. Participants were recruited through 10 local public rehabilitation centres, which are distributed over 8 different districts of Hong Kong. Written informed consents were obtained from participants prior to the enrollment.

**Fig. 2:**
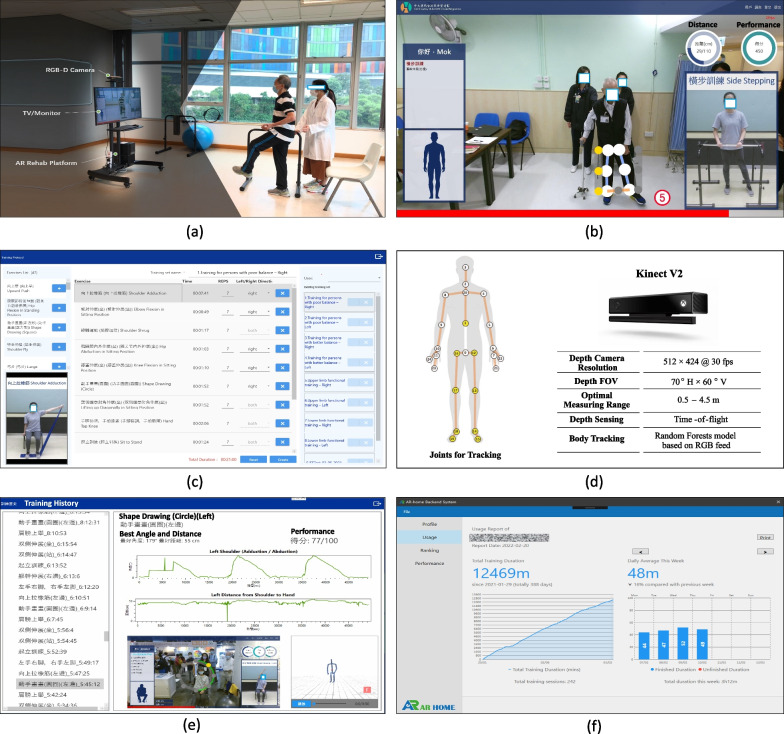
The AR rehabilitation system for virtual training. **a** System architecture and training scene; **b** Training in progress; **c** Training plan customization; **d** Body tracking joints and the Kinect sensor; **e** Real-time report; **f** Training statistics

### Participants

Chronic stroke survivors were selected with inclusion criteria: (1) age between 18 to 90 years who are diagnosed with ischemic brain injury, intracerebral haemorrhage shown by magnetic resonance imaging, or computed tomography after the onset of stroke; (2) with motor impairment in upper-limb, lower-limb, and/or balance; (3) have no or mild spasticity on the lower-limb or upper-limb assessed by Modified Ashworth Scale (MAS $\le$ 2); and 4) have sufficient cognition to follow the instructions provided by the therapists and the computer. The exclusion criteria included: (1) any additional medical or psychological condition that would affect their ability to comply with the study protocol, e.g., a significant orthopaedic or chronic pain condition, major post-stroke depression, epilepsy, artificial cardiac pacemaker/joint; (2) have severe shoulder/arm or hip/knee contracture/pain and; (3) Pregnant women. Potential participants were referred by their doctors or therapists, and screened at the nearest centres. A brief tutorial of using the AR training system was given, and then the participants were required to use the system as a test to see if they can follow the instructions and complete the exercises independently. Participants who failed in the test were excluded.

### Randomization and blinding

Randomization of group allocation occurred before the baseline assessment at centre level, in the way that participants who met the eligibility were first assigned to the nearest centre, and then randomly allocated to the AR-Centre or AR-Home group with a ratio of 1:1. The assignment of centre was due to the consideration of their limited mobility and the government’s regulations for prevention and control of disease during COVID-19. Baseline assessments were conducted after the assignments. This study was blinded at two levels. For the general objective of investigating whether AR rehabilitation worked, it was single blinded to assessors, because it was easy for recruiters, therapists and patients to see the difference from the usual care and was thus impossible to make this objective blind [34, 39, 48]. All assessors obtained their qualifications for the clinical assessments before joining the study. Furthermore, the objective in this study was not only to answer a question of whether AR training worked, but also to answer a more practical question of HOW it worked in a real and pandemic setting. The hypothesis was to use an integrated trial of human and virtual trainers. This was blinded to all recruiters, assessors, therapists, and patients.

**Fig. 3:**
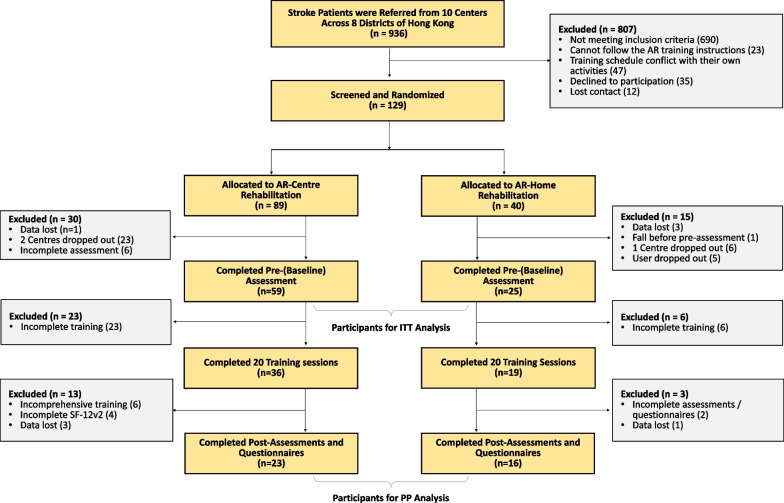
CONSORT diagram of this study. Centres dropped out because their services were limited due to the government’s regulation in the pandemic. Users dropped because of quarantine or not being able to return to Hong Kong. Assessment data were lost because the regular handover process was disrupted in the pandemic

### Intervention

Each participant received a standard trial consisting of 20 training sessions (2–5 sessions per week, 120 min per session) in consecutive 4–10 weeks. Each session consisted of 2–10 exercises selected by the trainer from a pool of 46 exercises. The selected exercises were tailored based on the participant’s condition and progress. The exercise pool was designed as much comprehensive as possible to cover the upper-limb (22 exercises) and lower-limb (7 exercises) motor functions, balance ability (11 exercises), or/and coordination (6 exercises). Each exercise was with a duration ranging from 3 to 23 min, and was determined by a set of parameters that can be adjusted to the individuals dynamically according to the participant’s performance.

The training was delivered in an integrated manner of a human PT/OT/Assistant trainer (called human trainer hereafter) and a AR rehabilitation system (called virtual trainer hereafter).

The two groups of AR-Centre and AR-Home were designed with different configurations of trainers to study the best way to integrate the AR training into usual care.

*AR-Centre* group: The training was conducted at the rehabilitation centres following the same procedure as their regular usual care, with 3/4 of each session delivered by a human trainer and the rest of 1/4 delivered by a virtual trainer.

*AR-Home* group: The training was conducted mainly at home (3/4 of each session) and delivered by the virtual trainers. However, at the beginning of each session, there was a short (half an hour or 1/4 time of a regular usual care session) in-centre/at-home training delivered by human trainers to make the participants familiar with the subjects and procedures. To this end, upon the recruitment, a human trainer was randomly assigned to a participant for tailoring her training plan according to the baseline assessment. While the participant was taking the training at home, the human trainer could monitor the training process remotely. Reports of the training were sent to the human trainer on a weekly basis. The human trainer could adjust the training plan based on the participant’s performance.

### The AR rehabilitation system for virtual training

As shown in Fig. 2, the AR rehabilitation system consists of a Microsoft Kinect V2 RGB-D camera for sensing the body movements of the patients, a TV/Monitor for displaying instructions and giving real-time assessments, and an AR rehabilitation software platform which has been implemented with the latest arts of AR and AI technology for delivering the training. The system is installed on a portable TV frame (NB MOUNT AVA 1500-60-IP) and is capable of communicating with our rehabilitation sever for synchronizing the latest training materials (videos, exercises, schedules, and training reports).

The system is able to deliver the exercises following the same procedure as in the usual care. The exercises performed by therapists are prerecorded. Every exercise starts by a brief voice introduction followed by a video demonstration with detailed steps. The participant will then follow the steps to do the exercise. Her body movements will be sensed into 3D skeletons, on the basis of which parameters such as the centre of Mass-COM, body parts, joint angles, reaching distances, speed and directions of the motions, and trajectory are evaluated. At the end of exercise, immediate feedback will be given as a report with the performance details and compared with her early performance. In addition, the participant can review her performance through the skeleton videos recorded during the exercise to locate the exact steps which she needs to pay more attention for further improvement. The performance details with the skeleton videos are synchronized to the cloud so that the human trainer could review her performance and adjust her training plan accordingly.

**Table 1:** Demographic and clinical characteristics of participant groups

Variables	AR-All	AR-Centre	AR-Home	p Value
	(n = 39)	(n = 23)	(n = 16)	
Age (yrs)^†^	63.54 ± 12.23	62.79 ± 13.79	64.61 ± 9.90	0.654
Gender (male/female)	21/18	10/13	11/5	0.218
Affected side (left/right)	10/29	7/16	3/13	0.480
Stroke type (isch./hemo.)	26/13	15/8	11/5	1.000
Time from Stroke Onset (yrs)^†^	3.51 ± 2.67	3.41 ± 2.72	3.67 ± 2.67	0.764
FMA-UE (max. 66)^†^	38.85 ± 19.98	40.09 ± 19.69	37.06 ± 20.91	0.648
FMA-LE (max. 34)^†^	18.64 ± 8.26	19.47 ± 8.18	17.44 ± 8.50	0.455
BBS (max. 56)^†^	40.90 ± 11.87	41.35 ± 13.05	40.25 ± 10.32	0.781
FAC (max. 6)^†^	4.54 ± 1.17	4.65 ± 1.19	4.38 ± 1.15	0.473
BI (max. 20)^†^	16.31 ± 2.73	N/A	16.31 ± 2.73	N/A
SF-12v2 PCS (max. 100)^†^	32.28 ± 12.25	32.75 ± 14.09	31.61 ± 9.39	0.779
SF-12v2 MCS (max. 100)^†^	40.60 ± 17.25	37.07 ± 19.29	45.68 ±12.68	0.127

*AR* augmented reality, *AR-All* AR-Centre and AR-Home, *AR-Centre* rehabilitation with AR Rehab in centre, *AR-Home* Telerehabilitation with AR Rehab at home, * FMA* Fugl-Meyer Assessment, *FMA-UE* FMA for Upper-Extremity, *FMA-LE* FMA for Lower-Extremity, *BBS* Berg Balance Scale, *FAC* Functional Ambulation Category, *BI* Barthel Index of Activities of Daily Living, *SF-12v2* The 12-Item Short Form Health Survey (PCS = Physical Component Summary and MCS = Mental Component Summary)

^†^Date are presented as mean ± SD

### Outcome measures

Participants received the pre-assessment within two weeks before the first training, and the post-assessment within two weeks after the last training. Assessments were conducted at centres for the AR-Centre group and at homes for the AR-Home group.

#### Primary outcome measures

The primary outcome measures focused on the motor function, balance, and functional ambulatory, which include (1) Fugl-Meyer Assessment with Upper Extremity and Lower Extremity (FMA-UE and FMA-LE); (2) Berg Balance Scale (BBS); (3) Functional Ambulation Category (FAC).

#### Secondary outcome measures

The secondary outcome measures included the assessments of (1) Quality of life (QOL): physical and mental health using the 12-Item Short Form Health Survey (SF-12v2); (2) User experience (UX) using questionnaires (designed based on UEQ-S); and (3) Activities of Daily Living (ADL) using Barthel Index (BI).

To evaluate the human effort required to deliver the training, the time of human training, the time of virtual training, the ratio of human training over the total training time, the number of human trainers required for each participant were calculated. The in-centre contact rate was calculated based on the numbers of PTs, OTs, training assistants, supporting staff, teammates the participant encountered at the centre. The numbers varied from centres and individuals. We used the numbers estimated by the PTs/OTs/Assistants and calculated the average across centres.

In addition, to study the clinical significance, we identified participants who were with improvement exceeding the Minimal Clinically Important Difference (MCID) or Minimally Detectable Change (MDC), on the basis of which we used the proportion of participants with clinical performance improvement as a supplementary measure for the efficacy of the training. More specifically, MCIDs reported in [49–51] were used for FMA-UE, BI, and SF12v2 PCS, and MDCs reported in [52], [53] were used for FMA-LE and BBS. For SF12v2 MCS, we used the $10\%$ of the maximum as the threshold, because there is no MCID/MDC reported in literature. Similarly, for FAC, the clinical significance was evaluated by whether a participant of limited mobility (baseline FAC$<4$) become an independent walker (post-assessment FAC$>=4$) [54].

#### Statistical analysis

The normality of all demographic, clinical variables was tested by using the Kolmogorov-Smirnov method or Shapiro-Wilk method and histogram inspection. To assess the group differences on the baseline data, we used Independent Sample *t* tests for numerical data with a normal distribution, and Mann-Whitney U tests for data with a skewed distribution. For categorical data, we used Pearson Chi-square tests or Fisher Exact tests. To assess the differences between pre- and post-assessments, Paired t-test was used for data with a normal distribution, and Wilcoxon Signed-Ranks tests were used for data with a skewed distribution.

All outcomes measures were analyzed using both per-protocol analysis (PP) and intention-to-treat principle (ITT), and missing data were dealt with the last observation carried forward method. All outcomes aim to evaluate whether the results demonstrate feasibility of assistance with virtual rehabilitation when integrated into the usual care in a real-world pandemic setting. Statistical results were reported with the effect size in confidence interval of $95\%$ (95% CI). Two-tailed levels of significance at 5% was used.

## Results

Participants were recruited from 10 centres across 8 districts of Hong Kong between September 2019 and August 2021. A total of 936 patients were screened, resulting in 129 from 10 centres. Eligible participants were randomly allocated to AR-Centre ($$n=89)$$ and AR-Home ($$n=40$$) groups. The numbers of participants in the two groups were with discrepancy, because during the pandemic, our capacity for home system deployment has been limited by both government’s regulation for disease control and the stock-out of the core hardware (Kinect sensors and TV monitors). Meanwhile, 45 patients withdrew after the baseline (pre-) assessment including 7 with incomplete baseline assessments, 5 were under the quarantine or were not able to return after leaving for abroad, 29 due to the limited service availability of the corresponding centres during the pandemic, and 4 with data lost in the disrupted (by the pandemic) handover process. This resulted in 84 participants who completed the baseline assessment. Data of these participants (AR-Centre: $$n=59$$, AR-Home: $$n=25$$) were used for ITT analysis. After that, 55 participants completed all 20 sessions, with 29 left with incomplete training because of the pandemic. There were 16 of them with incomplete data including 6 who missed from assessments/questionnaires, 6 who have only participated the upper limb training (instead of the comprehensive training of upper and lower limb, and balance), and 4 of data lost. This finally resulted in 39 participants with completed training and assessments. Data of these participants (AR-Centre: $$n=23$$, AR-Home: $$n=16$$) were used for PP analysis. The CONSORT diagram is shown in Fig. 3, and the demographic data of the participants is shown in Table 1.

Although group sizes of AR-Centre and AR-Home were with discrepancy, no significant difference was found on the baseline data in both PP and ITT analysis. It indicated that the selection and chance bias have been addressed by the randomization and blinding. In this section, we included the results of both PP and ITT analysis in tables. However, due to space limitation, we mainly referred to the statistics in PP analysis when describing the results in text. The statistical results of the two analysis methods were consistent to each other.

### Primary outcomes

The primary outcomes were shown as the first blocks in Table 2 (Tables 3) and  4 (Table 5). In Table 2, we presented the changes of all the participants without grouping to investigate the efficacy of the integrated intervention schemes. Significant difference of the post-assessment from the pre-assessment (baseline) were observed on both upper and lower extremity in FMA-UE ($3.64\pm 4.69$, $p=0.00002$) and FMA-LE ($3.92\pm 4.84$, $p=0.00001$), and on the balance in BBS ($2.23 \pm 5.70$, $p=0.019$). However, there was no significant difference on the functional ambulation in FAC ($0.22\pm 0.92$, $p=0.149$).

In Table 4 (Table 5), we presented the changes with comparisons between the AR-Home and AR-Centre groups. Significant differences from the baseline were observed on both groups, including the FMA-UE ($3.57\pm 4.93$, $p=0.0022$ for AR-Centre, $3.75\pm 4.46$, $p=0.0043$ for AR-Home), FMA-LE ($4.52\pm 5.48$, $p=0.0007$ for AR-Centre, $3.06\pm 3.75$, $p=0.0052$ for AR-Home). The improvement on balance was inconsistent on two groups with significant difference in BBS observed on AR-Centre ($1.87\pm 5.42$, $p=0.0438$), but insignificant on AR-Home ($2.75\pm 6.21$, $p=0.097$). Neither group was observed with significant difference on walk ability in FAC ($0.13\pm 0.87$, $p=0.48$ for AR-Centre, $0.34\pm 1.01$, $p=0.194$ for AR-Home). Comparing between the two groups, there was no significant difference on these parameters.

**Table 2:** Primary and secondary outcomes of all participants (AR-ALL, n=39) after 20 training sessions (PP analysis)

Outcome	Pre	Post	Difference	p Value
FMA-UE (max. 66)^†^	38.85 ± 19.98	42.49 ± 19.44	3.64 ± 4.69	0.00002**
FMA-LE (max. 34)^†^	18.64 ± 8.26	22.56 ± 7.78	3.92 ± 4.84	0.00001**
BBS (max. 56)^†^	40.90 ± 11.87	43.13 ± 10.30	2.23 ± 5.70	0.019*
FAC (max. 6)^†^	4.54 ± 1.17	4.76 ± 1.21	0.22 ± 0.92	0.149
BI (max. 20)^†^	16.31 ± 2.73	17.56 ± 2.53	1.25 ± 1.18	0.0007**
SF-12v2 PCS (max. 100)^†^	32.28 ± 12.25	37.10 ± 10.11	4.82 ± 9.11	0.002**
SF-12v2 MCS (max. 100)^†^	40.60 ± 17.25	45.54 ± 11.58	4.94 ± 13.05	0.023*

*FMA* Fugl-Meyer Assessment, *FMA-UE* FMA for Upper-Extremity, *FMA-LE* FMA for Lower-Extremity, *BBS* Berg Balance Scale, *FAC* Functional Ambulation Category, *BI* Barthel Index of Activities of Daily Living, *SF-12v2* The 12-Item Short Form Health Survey (PCS = Physical Component Summary and MCS = Mental Component Summary)

^†^Date are presented as mean ± SD

*p < 0.05; **p < 0.01

**Table 3:** Primary and secondary outcomes of all participants (AR-ALL, n=84) after 20 training sessions (ITT analysis)

Outcome	Pre	Post	Difference	p Value
FMA-UE (max. 66)^†^	36.70 ± 20.03	38.95 ± 20.16	2.25 ± 4.14	0.000003**
FMA-LE (max. 34)^†^	19.19 ± 7.21	20.88 ± 7.36	1.69 ± 4.46	0.00082**
BBS (max. 56)^†^	42.17 ± 11.13	43.52 ± 10.21	1.35 ± 4.14	0.004**
FAC (max. 6)^†^	4.69 ± 1.05	4.82 ± 1.06	0.14 ± 0.67	0.084
BI (max. 20)^†^	16.72 ± 2.54	17.60 ± 2.40	0.88 ± 1.13	0.0007**
SF-12v2 PCS (max. 100)^†^	32.15 ± 10.48	35.00 ± 9.81	2.85 ± 7.47	0.0008*
SF-12v2 MCS (max. 100)^†^	43.29 ± 17.55	46.04 ± 13.63	2.75 ± 11.40	0.030*

*FMA* Fugl-Meyer Assessment, *FMA-UE* FMA for Upper-Extremity, *FMA-LE* FMA for Lower-Extremity, *BBS* Berg Balance Scale, *FAC* Functional Ambulation Category, *BI* Barthel Index of Activities of Daily Living, *SF-12v2* The 12-Item Short Form Health Survey (PCS = Physical Component Summary and MCS = Mental Component Summary)

^†^ Date are presented as mean ± SD

*p < 0.05; **p < 0.01

**Table 4:** Primary and secondary outcomes of AR-Centre and AR-Home participants after 20 training sessions (PP analysis)

Outcome	AR-Centre (n = 23)			AR-Home (n = 16)			Mean	Group
	Pre	Post	Difference (p Value)	Pre	Post	Difference (p Value)	Difference (95% CI)	Difference p Value
FMA-UE^†^	40.09±19.69	43.65±19.41	3.57±4.93	37.06±20.91	40.81±20.01	3.75±4.46	− 1.563 (− 3.318	0.906
(Max. 66)			(0.0022**)			(0.0043**)	to 2.948)	
FMA-LE^†^	19.48±8.18	24.00±6.43	4.52±5.48	17.44±8.50	20.50±9.21	3.06±3.75	2.000 (− 1.741	0.361
(Max. 34)			(0.0007**)			(0.0052**)	to 4.659)	
BBS^†^	41.35±13.05	43.22±12.59	1.87±5.42	40.25±10.32	43.00±6.02	2.75±6.21	− 1.3125 (− 4.677	0.641
(Max. 56)			(0.0438*)			(0.097)	to 2.916)	
FAC^†^	4.65±1.19	4.78±1.17	0.13±0.87	4.38±1.15	4.72±1.32	0.34±1.01	− 0.281 (− 0.826	0.485
(Max. 6)			(0.480)			(0.194)	to 0.400)	
BI^†^	N/A	N/A	N/A	16.31±2.73	17.56±2.53	1.25±1.18	N/A	N/A
(Max. 20)						(0.0007**)		
SF-12v2 PCS^†^	32.75±14.09	37.58±10.37	4.83±9.79	31.61±9.39	36.42±9.99	4.81±8.35	0.888 (− 6.078	0.996
(Max. 100)			(0.027*)			(0.036*)	to 6.105)	
SF-12v2 MCS^†^	37.07±19.29	45.07±13.16	8.01± 14.87	45.68±12.68	46.21±9.18	0.53±8.47	8.878 (− 0.877	0.078
(Max. 100)			(0.017*)			(0.807)	to 15.837)	

*FMA* Fugl-Meyer Assessment, *FMA-UE* FMA for Upper-Extremity, *FMA-LE* FMA for Lower-Extremity, *BBS* Berg Balance Scale, *FAC* Functional Ambulation Category, *BI* Barthel Index of Activities of Daily Living, *SF-12v2* The 12-Item Short Form Health Survey (PCS = Physical Component Summary and MCS = Mental Component Summary)

^†^Date are presented as mean ± SD

*p < 0.05; **p < 0.01

**Table 5:** Primary and secondary outcomes of AR-Centre and AR-Home participants after 20 training sessions (ITT Analysis)

Outcome	AR-Centre (n = 59)			AR-Home (n = 25)			Mean	Group
	Pre	Post	Difference (p Value)	Pre	Post	Difference (p Value)	Difference (95% CI)	Difference p Value
FMA-UE^†^	36.95±19.87	39.29±20.28	2.34±3.98	36.12±20.80	38.16±20.26	2.04±4.57	1.480 (− 1.677	0.760
(Max. 66)			(0.00003**)			(0.035*)	to 2.275)	
FMA-LE^†^	20.32±6.39	21.95±6.14	1.63±4.85	16.52±8.39	18.36±9.31	1.84±3.45	2.320 (− 2.343	0.843
(Max. 34)			(0.0005**)			(0.013*)	to 1.917)	
BBS^†^	43.00±11.97	44.14±11.58	1.13±3.69	40.20±8.74	42.08±5.79	1.88±5.09	0.192 (− 2.716	0.452
(Max. 56)			(0.022*)			(0.077)	to 1.221)	
FAC^†^	4.76±1.02	4.85±1.00	0.08±0.60	4.52±1.12	4.78±1.21	0.26±0.83	− 0.14 (− 0.495	0.122
(Max. 6)			(0.317)			(0.114)	to 0.144)	
BI^†^	N/A	N/A	N/A	16.72±2.54	17.60±2.40	0.88±1.13	N/A	N/A
(Max. 20)						(0.0007**)		
SF-12v2 PCS^†^	31.93±11.36	34.58±10.34	2.66±7.74	32.67±8.23	35.98±8.57	3.31±6.95	2.090 (− 4.217	0.718
(Max. 100)			(0.011*)			(0.026*)	to 2.918)	
SF-12v2 MCS^†^	42.14±19.34	45.63±15.05	3.49± 12.76	46.00±12.29	46.99±9.70	0.99±7.14	8.044 (− 2.910	0.360
(Max. 100)			(0.040*)			(0.495)	to 7.919)	

*FMA* Fugl-Meyer Assessment, *FMA-UE* FMA for Upper-Extremity, *FMA-LE* FMA for Lower-Extremity, *BBS* Berg Balance Scale, *FAC* Functional Ambulation Category, *BI* Barthel Index of Activities of Daily Living, *SF-12v2* The 12-Item Short Form Health Survey (PCS = Physical Component Summary and MCS = Mental Component Summary)

^†^ Date are presented as mean ± SD

*p < 0.05; **p < 0.01

### Secondary outcomes

The secondary outcomes were shown as the second blocks in Table 2 (Tables 3) and 4 (Table 5). In Table 2, significant improvement was observed on all secondary parameters when considering all participants as a whole, with BI ($1.25\pm 1.18$, $p=0.0007$), SF-12v2 PCS ($4.82\pm 9.11$, $p=0.002$), and SF-12v2 MCS ($4.94\pm 13.05$, $p=0.023$).

In Table 4 (Table 5), the BI was missing from the AR-Centre while was observed with significant difference on the AR-Home group ($1.25\pm 1.18, p=0.0007$). Consistent improvement was observed on two groups in SF-12v2 PCS ($4.83\pm 9.79$, $p=0.027$ for AR-Centre, $4.81\pm 8.35$, $p=0.036$ for AR-Home). The observation in SF-12v2 MCS was also inconsistent which is significant on AR-Centre ($8.01\pm 14.87$, $p=0.017$) and insignificant on AR-Home ($0.53\pm 8.47$, $p=0.807$). Comparing between two groups, there was no significant difference observed on the secondary outcomes, which is consistent with the primary outcomes.

The training effort and contact rate were presented in Table 6. By integrating AR virtual training, the trial could save the human effort from the regular usual care by $33.19\%$ when considering all participants as a whole (AR-All), by $19.05\%$ within the AR-Centre group, and by $57.78\%$ within the AR-Home group. The in-centre contact rate could be reduced by 30.75–72.30% (2.46–7.23 persons) within AR-All, 0.00–60.00% (0–6 persons) within AR-Centre, and 75.00–90.00% (6–9 persons) within AR-Home.

**Table 6:** The human effort for delivering the training and the in-centre contact rate of participants

Variables	Regular	AR-All	AR-Centre	AR-Home	p Value
	Usual care	(n = 39)	(n = 23)	(n = 16)	(Centre vs. Home)
Duration of training (wks)^†^	10.00	14.22 ± 7.50	16.92 ± 8.49	10.35± 3.07	0.005**
Time of human training (hrs)^†^	40.00	32.00 ±18.74	41.74 ± 17.54	17.99 ± 9.13	0.000016**
Time of virtual training (hrs)^†^	0.00	15.89 ± 11.50	9.82 ± 3.10	24.62± 13.53	0.000011**
Ratio of human training (%)	100.00	66.81	80.95	42.22	0.000338**
Human trainers required (#persons)	2.86	N/A	1.86	1.14	
In-Centre contact rate (#persons)	8 – 10	2.77 – 5.54	4 – 8	1 – 2	

The Human trainers required and In-Centre contact rate vary from centres. The numbers presented are the averages across centres

^†^ Date are presented as mean ± SD

*p < 0.05; **p < 0.01

**Table 7:** User experience of participants and centre staff

Questions for participants	Rate (0–5)
I am satisfied with the system	4.33
I feel the system is easy to use	4.00
I am satisfied with the virtually delivered training	4.15
There is an improvement of my upper extremities	3.74
There is an improvement of my lower extremities	3.65
There is an improvement of my balance	3.74
I would like to use this device at home to continue my training	3.89
I would recommend this equipment to others	3.96
I am satisfied with the training program	4.29
I will participate in similar programs again in the future	4.41
I would like to use more of the newly developed functions	4.44

Questions for human trainers (PTs/OTs)	Rate (0–5)
I feel the system is easy to use	4.36
I feel I have mastered how to use the system	4.18
I would like to use the system more frequently in our training programs	4.00
I think the system is effective for stroke patients	3.82
I understand more about the rehabilitation systems through this program	3.82
I can use the system independently without the assistance of the technicians	4.09
I am satisfied with this program	4.00

**Table 8:** The proportions of participants with performance improvement exceeding MCIDs/MDCs

Outcome	MCID/MDC	PP analysis (n=39)		ITT analysis (n=84)	
		AR-Centre (n = 23)	AR-Home (n = 16)	AR-Centre (n = 59)	AR-Home (n = 25)
		% Post-pre > MCID	% Post-pre > MCID	% Post-pre > MCID	% Post-pre > MCID
FMA-UE	5.25 (MCID) [49]	34.78%	37.50%	13.56%	12.00%
FMA-LE	3.57 (MDC) [52]	52.17%	62.50%	20.34%	40.00%
BBS	2.7 (MDC) [53]	43.48%	37.50%	16.95%	24.00%
FAC	4$^*$	4.35%	6.25%	1.69%	4.00%
BI	1.85 (MCID) [50]	N/A	37.50%	N/A	24.00%
SF-12v2 PCS	2.5 (MCID) [51]	47.83%	68.75%	18.64%	44.00%
SF-12v2 MCS	10$$^{**}$$	30.43%	12.50%	11.86%	8.00%

*FMA* Fugl-Meyer Assessment, *FMA-UE* FMA for Upper-Extremity, *FMA-LE* FMA for Lower-Extremity, *BBS* Berg Balance Scale, *FAC* Functional Ambulation Category, *BI* Barthel Index of Activities of Daily Living, *SF-12v2* The 12-Item Short Form Health Survey (PCS = Physical Component Summary and MCS = Mental Component Summary), *MCID* minimal clinically important difference, *MDC* minimally detectable change

$^*$ A threshold of 4 was used. A participant’s performance gain on FAC is clinically significant if and only if she was with limited mobility in the baseline assessment (FAC $<4$) and become an independent walker after the trials (post-assessment FAC $\ge$4) [54]

$$^{**}$$ 10% of the maximum was used as the threshold

The results of the user experience were shown in Table 7. The rate for user acceptance is 81.09% and 80.77% on average among participants and human trainers, respectively.

To measure the clinical significance, the proportions of participants with performance improvements exceeding the MCIDs/MDCs/$10\%$ max. were shown in Table 8. In the AR-Centre (AR-Home) group, the proportions were 34.78% (37.50%) on upper limb, 52.17% (62.50%) on lower limb, 43.48% (37.50%) on BBS, 4.35% (6.25%) on FAC, N/A (37.50%) on BI, 47.83% (68.75%) on SF-12v2 PCS, and 30.43% (12.50%) on SF-12v2 MCS, respectively.

### Adverse events

There is no study-related adverse events reported during the process of the rehabilitative intervention in either the AR-Centre or AR-Home group.

## Discussion

The results give clear evidence that the trial integrated with the AR training is feasible in a real-world setting to fulfil the training goals. In terms of the intervention, significant improvement on the upper/lower extremity (in FMA-UE/LE), balance (in BBS), and quality of life (in SF-12v2 PCS/MCS) are observed. The improvement is comprehensive when compared with those of the previous studies which focus on specific functions. This is also different from the conclusion made in [47] that the majority of AR/VR intervention methods are reported with no significant improvement. However, it is not surprising because the essential difference of our integrated training from those methods is that the AR training is designed as an extension of the PT/OT rather than video games or training for specific functions. This makes the training plans are more practical.

First of all, the training plans are tailored to the same extent of that of the usual care. The plans can thus dynamically address (and be adjusted to) the specific needs of the participants, and provide a comprehensive intervention by paying a balanced attention on the participants’ functions. Secondly, the integrated scheme draws a boundary between the training efforts on the teaching and practising. It makes the human trainers can focus more on the teaching and explaining of the trials. This part needs more perceptual intelligence that is still an open question for AR research. Meanwhile, the integrated scheme leaves the AR training mainly for the practising part, in which the key is the repetition of what being taught. The human trainers are thus released from this time-consuming part and their general availability to participants are maximized. The efficacy of the balanced efforts is evident in Table 6. It shows that the 20 training sessions were completed in AR-Home groups within $61.17\%$ of the time of that in AR-Centre group (10.35 wks vs. 16.92 wks) with the same significant improvement observed (see Table 4). The reason is that the AR-Home participants are able to receive the training sessions at least twice every week (which reaches the same level of the regular care before the pandemic) because of the increased availability of human trainers. At the same time, without the dependence on the human trainers, the participants are self-driven to practise 2.51 times longer than those in AR-Centre group (24.62 hrs vs. 9.82 hrs), so that their training effect is still ensured with the compact schedule.

This leads to the reduction of contact rate as well. It makes the training possible even during the COVID-19 regulation from February 2020 to February 2021 in Hong Kong when the government imposed strict restriction on group gathering of more than 4 (sometimes 2) persons. The rehabilitation services of seven centres were suspended at that time because it was difficult to deliver the training with the conventional scheme (which usually requires a team of a PT/OT and a few assistants). By contrast, 3 centres delivering our integrated program were still open for the services during that period. The AR-Home group was not affected because the training could be conducted at home by the participants with remote assistance of human trainers. Our records show that the average practice time of AR-Home group reached 2.64 hrs/week, which is 2.29 times longer than that of the less strict periods. Several participants reveal in our random interviews that their worries of missing the recovery period due to the regulation had drove them to conduct more self-training. The AR system installed at their homes made it happen.

**Fig. 4:**
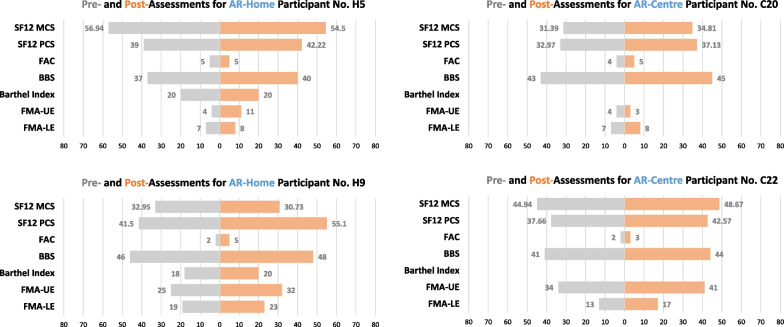
The pre- and post-assessment details of participants. No. H5 (H9) and No. C20 (C22) are from AR-Home and AR-Centre groups, respectively

The feasibility of the integrated scheme also comes from its user acceptance. Despite the fact that the integrated scheme was one of a few possible solutions during the COVID-19, the results on the Table 7 show AR training is generally accepted by both participants and human trainers. One reason is that the AR training is an extension of their usual care so that the participants can work with the virtual trainers seamlessly. In addition, the design of the AR training with focus on practice has simplified the use of the system, because the training content is already delivered by the human trainers. The gait independence (FAC) is a measure in which significant improvement is not observed. It might result from our selection criteria which requires the participants are capable of using the AR training system independently. This requirement is an indirect filter that excludes the candidates with baseline below FAC 4 “Dependent, Supervision” (Ambulation occurs on level surfaces without manual contact of another person. Requires stand-by guarding of one person because of poor judgment, questionable cardiac status, or the need for verbal cuing to complete the task.). Consequently, there is a limited room for improvement in FAC among our participants. Nonetheless, this helps clarify that target users of this type of AR training systems.

The BBS is a measure with inconsistent observations (significant for participants as a whole or in the AR-Centre group, not significant in the AR-Home group). This might be due to the human trainers’ concern of safety that it is dangerous for the participants to conduct the balance related training independently at home. Therefore, they intentionally reduced the amount of balance training for those in the AR-Home group, which may result in the less significant improvement. However, the statistical evidence in both PP and ITT do not support this hypothesis well, because there is no significant difference on BBS between the AR-Centre (with full training) and AR-Home (with reduced mobility training). The evidence is too weak to make the conclusion because the two groups are with different configurations of trainers. To answer this question, we need to conduct a specific experiment in the future by aligning the configurations.

The SF-12v2 PCS is significant improved (for participants of all groups). However, the SF-12v2 MCS is with inconsistent observations (significant for participants as a whole or in the AR-Centre group, not significant in the AR-Home group). This is due to the fact that AR-Home users have less (face-to-face) communication with their PTs/OTs and friends than those of the AR-Centre, which is critical to how they feel about their quality of life (QOL) on mental well-being. In Fig. 4, we compare the assessment details of 4 participants. The AR-Home participant No. H5 (H9) is with similar improvement (on all parameters except SF-12v2 MCS) with that of the AR-Centre participant No. C20 (C22). This is an indication that similar rehabilitation efficacy of their physical functions have been archived. However, the inconsistent observations on SF-12v2 MCS indicate that AR-Centre participants may feel better about their mental QOL. By comparing the improvement of BI which is an index for activities of daily living (ADL), this is more evident that the participants’ feeling about the improvement is not necessarily consistent with the facts. Again, we can learn from the results that mental care should be improved in the future design.

In this study, we include the comparison to MCID or MDC to investigate the clinical significance of the improvements. The method is indeed rarely used in AR rehabilitation for chronic stroke recovery (i.e., it is only used in 1 out of 31 previous studies published in recent 10 years).

The reason might be that the chronic stroke recovery is a long-term, slow, and non-linear process [55–59] which may span years. A study with a period of several weeks or months thus has less chance to observe clinically significant improvement. However, the results in Table 8 shows more than 1/3 of our participants have achieved improvements exceeding the MCIDs or MDCs. The proportion on SF12v2 PCS is as high as $68.75\%$ in PP analysis. This is encouraging. We believe it is another indication of the efficacy of the integrated trials. Nonetheless, this is not conclusive, given the fact that the sample size is still small. Experiments on a larger scale are expected for further verification.

### Limitations

While encouraging results have been observed, there are several limitations. First of all, the attrition in this study is high. It is mainly due to the limited availability of on-site services at centres during the pandemic and the quarantine of participants. It reflects one of the nature of such studies when facing a force majeure like COVID-19 [60]. One possible solution is to recruit more home users which rely less on the on-site services. However, it raises the challenges to system deployment at the same time and might be beyond the capacity of a research lab. We may need partners from industry in the future when the study scales up.

Furthermore, we have seen that the human trainers reduced the balance training because of the safety concern. It reveals the limitation of AR rehabilitation for mobility-impaired individuals. In a more general sense, this is in fact an open question for most of the Tele-rehabilitation systems, but not sufficiently discussed because a majority of previous studies are conducted in a lab setting (as shown in Fig. 1). Another closely related limitation is that mobility training often requires equipment that is rarely available at home. Our system has made the training available to the maximum extent by including exercises in sitting positions to move the legs or shift the body. For those who have difficulty to travel to hospitals or clinics, this makes the training possible for them better than having nothing at all. However, there is still a gap from the training using specific equipment. A better design of experiments/systems is needed in the future to address this issue.

Moreover, some home participants have raised the privacy concerns to the cameras, although we technically only save the 3D skeletons. This might be addressed in the future when non-RGB motion sensors (e.g., event cameras, WiFi-based motion sensors) became a mature technology.

In addition, frequency of training of two groups are different from each other, although we have fixed the training to 20 sessions. This might contribute to the efficacy of training and needed to be further investigated in the future.

## Conclusions

We have conducted a feasibility study to investigate the efficacy of our rehabilitation programs which integrate the AR training into the participants’ usual care. The results showed that the integrated mode can significantly reduce the human (PTs/OTs/Assistants) trainers’ effort on the training as well as to reduce the contact rate during COVID-19 pandemics. The AR training system turns the time-consuming the monotonous practising part of the trials into an automated process which can be conducted by the participants independently. This also releases the human trainers so that they can focus more on the teaching and explaining part. We believe this is a practical rehabilitation mode with well-balanced human–machine coordination which is effective and efficient during the pandemics.

## Data Availability

All data generated or analysed during this study are included in this published article. Information on this clinical trial can be found at: https://clinicaltrials.gov/ct2/show/NCT04638218.

## Abbreviations

AR: Augmented Reality
AR Rehab: Our Augmented Reality Rehabilitation System
AR-All: AR-Centre and AR-Home
AR-Centre: Rehabilitation with AR Rehab in centre
AR-Home: Telerehabilitation with AR Rehab at home
FMA-UE: Fugl-Meyer Assessment for Upper Extremit
FMA-LE: Fugl-Meyer Assessment for Lower Extremit
BBS: Berg balance scale
FAC: Functional ambulation category
FAC: Functional ambulatory category
BI: Barthel Index of Activities of Daily Living
ADL: Activities of daily living
SF-12v2: The 12-item short form health survey
SF-12v2 PCS: Physical component summary of SF-12v2
SF-12v2 MCS: Mental component summary of SF-12v2
UX: User experience
PTs: Physiotherapists
OTs: Occupational therapists
VR: Virtual reality
HMD: Head-mounted display
EMG: Electromyography
FES: Functional electric stimulation
Lab: Laboratory
H: Home
MAS: Modified Ashworth Scale
COM: Centre of mass
QOL: Quality of life
UEQ: User Experience Questionnaire
UEQ-S: A short 8-item version of the UEQ
MCID: Minimal clinically important difference
MDC: Minimally detectable change

## Footnotes

1 Although there are several studies [14, 17, 19, 22, 34, 36, 42] are under hospital/centre setting, they were designed for research rather than daily rehabilitation programs. In [17], the experiment was conducted under home setting, but the study was conducted with only 2 users.
